# Towards sustainable land use: A geospatial analysis of soil moisture content in a mining-induced degraded landscape of Ghana

**DOI:** 10.1007/s10661-026-14989-9

**Published:** 2026-01-19

**Authors:** Joseph Oduro Appiah, Richard Larbie

**Affiliations:** 1 Department of Geography, Bucknell University, One Dent Dr., Lewisburg, PA 17837 USA; 2 Department of Geography and Rural Development, Kwame Nkrumah University of Science and Technology, Private Mail Bag (PMB), Kumasi, Ghana

**Keywords:** Surface mining, Volumetric water content, Soil moisture, Regression analysis, Environmental modeling, GIS

## Abstract

Mining activities in tropical savanna regions can severely disrupt soil structure and vegetation, yet the factors influencing soil moisture content in post-mining landscapes are not fully understood. This study focused on the factors associated with soil moisture in a mining-induced degraded landscape. This study hypothesized that there is no significant relationship between soil moisture and the presence of open grasses, open shrubs, and closed shrubs. Through a field survey, soil moisture data were collected from an abandoned, unreclaimed mine land in Ghana. Ten univariate and two multivariable GIS-based generalized linear regression models were constructed to assess the relationship between soil moisture and several independent variables, including the presence of vegetation. The results show that the presence of open grasses, open shrubs, and closed shrubs significantly explains 52% of the variation in soil moisture (R^2^ = 0.520, p < 0.05). Soil moisture is 18.04%, 15.56%, and 14.30%, significantly higher in open grasses, open shrubs, and closed shrubs, respectively, compared to bare soil (p < 0.05). While soil temperature significantly predicts soil moisture values in the univariate model, its statistical significance is masked by factors, including open grasses, open shrubs, closed shrubs, elevation, slope, topographic wetness index, north-facing direction, and south-facing direction, in the multivariable model. Our results suggest that in savanna areas where moisture-laden soil is essential for reclaiming mine-degraded landscapes, and enhancing the likelihood of achieving Sustainable Development Goal 15, it is necessary first to improve grass cover to moisten the soil, followed by planting tree- and non-tree shrubs.

## Introduction

The earth system would be able to support humans with ecosystem services (e.g., food, water, etc.), provided environmental quality is maintained, and environmental decision-making ensures sustainable land use. However, in recent years, the need for resources for socio-economic development has led to the creation of ecological footprints which would likely degrade the ability of the earth to function as a support system continuously. Activities such as mining, logging, and agriculture have had profound negative impacts on environmental resources, including soil, water, and plant life. The recent rush for earth’s precious mineral resources has led to an increase in the mining of gold and diamonds, and this has exacerbated the rate at which land is being degraded in mining communities across the globe (Bazillier & Girard, 2020; Hammond et al., 2007; Hart, 2014; Nyamunda & Mukwambo, 2012). Thus, with such a rush for gold and diamonds, important earth materials (e.g., soil nutrients, soil organic matter, soil moisture, etc.) needed to support plant growth have been degraded, especially in areas where surface mining activities are not regulated, and restoration efforts are minimal (Bazillier & Girard, 2020).

Mining has contributed to the degradation of landscapes, and it casts doubt on the sustainability of the activity. Socio-economically, mining activities are beneficial, but the negative environmental impacts associated with the activity are enormous. For instance, mining activities have contributed to soil degradation (Adeoye, 2016; Basir-Cyio et al., 2012, 2020; Caballero Espejo et al., 2018; Grimaldi et al., 2015; Salami et al., 2003). In some areas, including Ghana, mining has been associated with water pollution (Armah et al., 2013; Casso-Hartmann et al., 2022; Minnaar, 2020; Ning et al., 2011) and to a greater extent, forest degradation (Caballero Espejo et al., 2018; Diringer et al., 2020; González-González et al., 2021; Peterson & Heemskerk, 2001; Siqueira-Gay & Sánchez, 2021; Swenson et al., 2011). Thus, previous studies have focused on a variety of land use impacts of mining, including impacts on water resources, vegetation, and soil (Caballero Espejo et al., 2018). However, over the past few years, due to the rampant loss of vegetation and biodiversity, the environmental impacts of mining and associated land degradation have become a major issue of concern. Also, the need to achieve Sustainable Development Goal (SDG) 15, which seeks to, “protect, restore and promote sustainable use of terrestrial ecosystems, sustainably manage forests, combat desertification, and halt and reverse land degradation and halt biodiversity loss,” is a critical global environmental concern. Considering this goal, a study examining how vegetation influences the soil moisture content of mining-induced bare lands is partly integral to understanding land degradation, land reclamation, and achieving SDG 15.

The creation of bare surfaces through the removal of vegetation would likely thwart post-mining vegetation regeneration efforts, especially in areas where organic matter is low and nutrients and moisture needed in soil to ensure plant growth are also minimal (Walker & Powell, 2001). Soil moisture, for instance, is one of the most important environmental factors that determines vegetation composition, structure, and functioning of terrestrial ecosystems (Raduła et al., 2018). Thus, the state of moisture content of the soil is very important, especially on abandoned mine sites where genuine and renewed efforts are needed to reclaim degraded lands. For land reclamation of soil to be deemed effective, a healthy biodiversity of an ecosystem balanced in terms of physical, chemical, and biological components is required (Shrestha, 2011). However, disturbances such as surface mining disturb the ecosystem balance through vegetation removal, soil removal, overburdening of soil (by excavators), and permanent surface and subsurface disruption (Shrestha, 2011). To support conservation efforts and reverse land degradation as envisioned through SDG 15, there is a need for studies that assess and monitor the state of the bare land resulting from surface mining activities. Consequently, this study assessed the relationship between soil moisture and open grasses, open shrubs, closed shrubs, and other control variables in an area within the Talensi District of Ghana. Whereas a study from such an area covers a landscape level, it has global implications. For instance, assessment and reclamation of degraded lands, several degraded ones at a landscape level, would contribute to the global efforts in achieving SDGs, including ensuring sustainable use of land for human activities and halting biodiversity loss.

The focus of previous studies justifies this study, as research involving mining and land degradation in most tropical environments focuses highly on two main areas of environmental assessments, noted as follows. First, previous studies have focused on how mining activities are degrading forest cover in protected landscapes (Asiamah, 2020; Boadi et al., 2016; Hilson & Nyame, 2006; Moraes et al., 2002; Muhire et al., 2021; Siqueira-Gay et al., 2020). These studies, which focused on the tropical rainforests of Africa (Ghana and Rwanda) and Latin America (Brazil) found that mining activities are degrading forests in forest reserves. Second, previous studies have focused on mining activities and the pollution of agricultural land (Asare, 2021; Limei et al., 2008; Mihalík et al., 2011) as well as their impacts on food security, including competition for land for food production and mining activities (Moomen & Dewan, 2015; Ocansey, 2013). A few of these studies have focused on mining activities and their impacts on soil moisture (Cao et al., 2022; Liu & Ma, 2016; Wang et al., 2022). For instance, through a previous study, it is known that when moving from wet to dry conditions, the soil in the disturbed area dried faster than the soil in the undisturbed area after the soil gets wet, but this study focused on disturbed land on the Chinese Loess Plateau, where surface disturbances are mainly from coal mining (Cao et al., 2022). However, our current study provides a different perspective on the relationship between land use and environmental degradation by focusing on a tropical savanna area where excessive heat and drought are likely to exacerbate the lack of soil moisture on bare grounds (Usman & Nichol, 2020; Usman et al., 2018), an area of land predominantly exposed because of surface mining activities. Does the presence of scant vegetation types (isolated grass, open shrubs, closed shrubs) influence the volumetric water content of soil? This novel question has been explored in the current study.

In this study, our goal was to model the relationship between soil moisture (represented by volumetric water content), open grasses, open shrubs, closed shrubs, and other control variables. Based on the research gap identified and the goal of the study, the following hypotheses were set to direct the study. The hypothesis was tested as part of the study to determine the relationship between soil moisture, vegetated, and non-vegetated spaces on the abandoned mining landscape. In formulating the hypothesis, a null hypothesis was stated; there is no significant relationship between soil moisture, open grasses, open shrubs, and closed shrubs in the abandoned mined area. The outcome of the study would serve as a guide for land managers who are tasked with the rehabilitation of land that has been used for mining over the past years. Most importantly, this study is relevant because it provides information regarding the need to improve degraded lands for alternative land uses in the future. Additionally, this study is important because it highlights the need to maintain vegetation cover on landscapes in an era where some areas are getting drier than before because of global warming and associated evapotranspiration.

## Methodology

### Study area

**Fig. 1:**
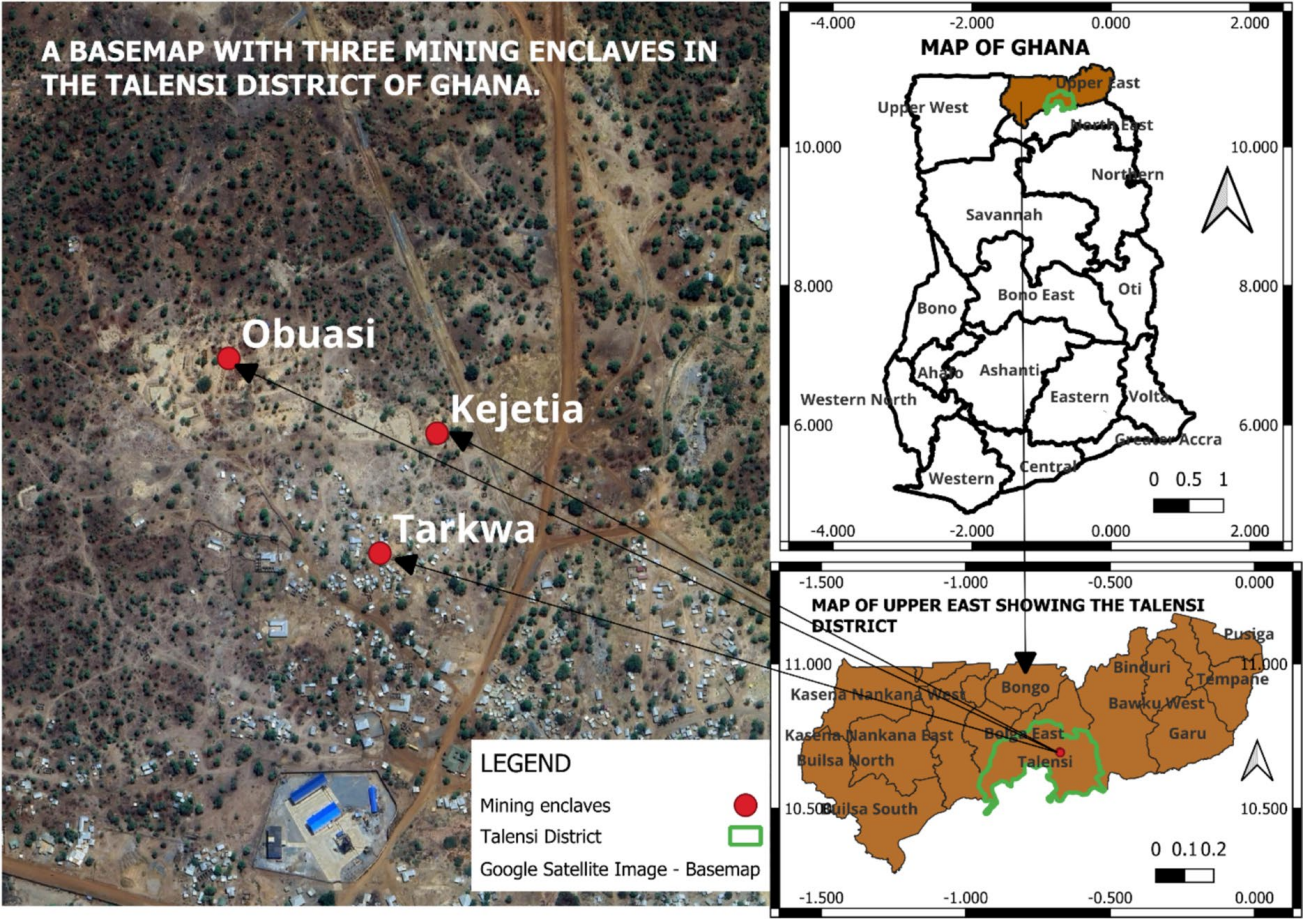
Study area in Talensi District. (The figure shows the study area, three mining enclaves, the Upper East region, and Ghana.)

**Fig. 2:**
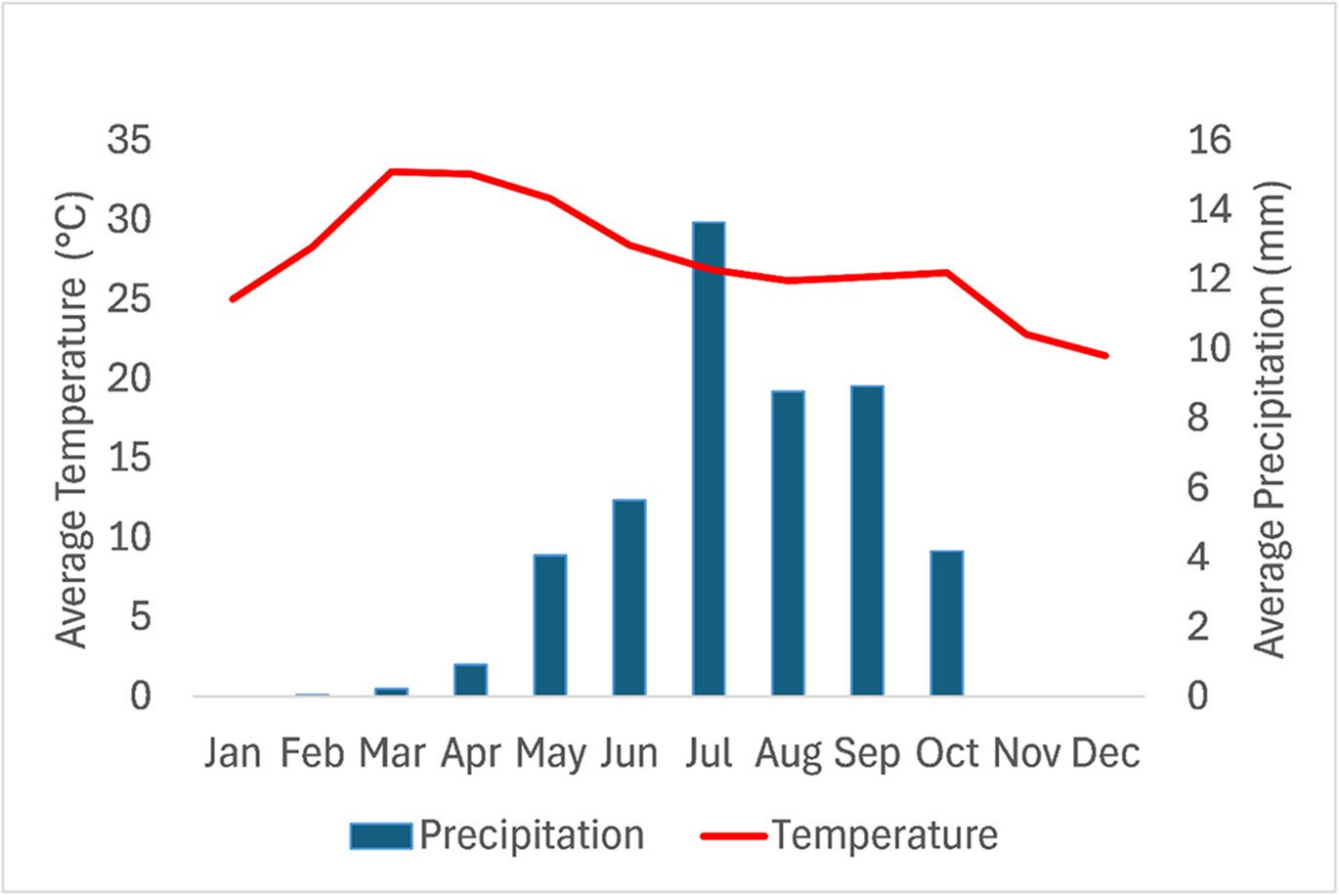
Average temperature and precipitation in the Talensi District of Ghana, 2024

The study area (see Fig. 1) is located in the Talensi District of Ghana, an area where human settlements developed principally due to the booming gold mining business. The study area is characterized by a single-peak unimodal rainfall pattern, with its rainy season and prolonged dry season usually dominated by the harmattan winds occurring from May to October and November to March, respectively. The climate of the area is classified as tropical savanna, with average annual rainfall between 850 mm and 1,050 mm, and its diurnal temperatures range from about 20 °C in the early morning to over 40 °C in the afternoon during the dry season. Average annual temperatures hover around 28 °C to 32 °C (see Fig. 2, for instance, showing typical monthly average temperature and precipitation). The soil is predominantly shallow, lateritic, and sandy loam, often low in organic matter and susceptible to erosion. Vegetation in the district is mainly Guinea Savannah woodland, characterized by scattered drought-resistant trees like baobab (Adansonia digitata) and shea (Vitellaria paradoxa), interspersed with grasses, with human activities, particularly farming and mining, having contributed to the degradation of both soil and vegetation cover in the area.

This area was selected for the study based on the fact that surface mining has occurred there, and there are several piles of earth materials, bare grounds, and mining holes. Also, this area was selected because it has been abandoned without any reclamation efforts after the surface mining activities. Thus, assessing the relationship between the land cover types and soil moisture would provide spatial information for land reclamation and conservation efforts in the area and many other locations where lands have been abandoned after surface mining activities. It is important to note that not every part of the landscape in the area has been used for surface mining activities. The study area is close to the three mining enclaves formed around 1995 (Van de Camp, 2020). These include the enclaves of Kejetia (named after a big market center in Kumasi), Obuasi (named after the mining town where AngloGold Ashanti is located), and Tarkwa (named after the mining town of Tarkwa). These enclaves are within the village lands of Datuku and Gbani. Human settlements in the enclaves are made up of approximately 300 people per enclave. People in these areas settled mainly because of their participation in mining activities (Adam et al., 2022; Van de Camp, 2020).

### Data collection procedures

While this study did not involve any human subjects, our ability to collect data at the study areas was determined by community members’ willingness to allow us (the researchers) to get access to their abandoned mining land. Also, we would like to mention that community members were agitated even after allowing us to collect data because of the suspicion that we were working for the Government of Ghana to stop illegal mining activities. The data for this study included volumetric soil water content (VSWC), soil temperature, and vegetation cover types collected at randomly generated point locations. The randomly generated points were created using the Create Random Points tool in ArcGIS Pro. Additionally, a digital elevation model (DEM) with a resolution of 30 m by 30 m was utilized. In ArcGIS Pro, 220 random points and grids were created with an Environmental Research Institute (ESRI) image as a background image. These point locations indicated by a grid and a point were assigned geographic coordinates and loaded into a E1 real-time kinematic (RTK)/global positioning system (GPS) (SingularXYZ, No. 599 Gaojing Road, 201,702 Shanghai, China). The E1 RTK/GPS has a ± 2.5 cm positioning accuracy. With E1, random locations were sought on the landscape, and measurements of soil moisture and soil temperature were recorded. In this study, vegetation cover types were recorded at the same random locations as other variables (e.g., soil moisture, soil moisture, etc.). Thus, the procedure for sampling for vegetation types was done to match that of the other variables included in the models constructed in this study.

Volumetric water content and soil temperature data were collected using FieldScout TDR 350 (Spectrum Technologies, Aurora, IL, USA). Vegetation data were collected during a field survey on July 7, 2024, in the study area. Vegetation types, open grasses, open shrubs, and closed shrubs were identified during the field survey. Open grasses are non-tree and dominant in the landscape. These grasses are narrow-leaved, and cover parts of the soil, leaving some part of the soil still visible. Open shrubs are tree and non-tree vegetation with broader leaves than grass and still partly cover the soil. Closed shrubs are tree and non-tree vegetation, and they have broader leaves than open shrubs and completely cover the soil. This categorization of the vegetation cover types was adapted from a previous study in a savanna region of Botswana (see Van De Griend & Owe, 1993). The DEM dataset was collected from the United States Geological Survey archives.

In this study, the VSWC, sometimes expressed as a measure of the percentage of water in the soil, was used as a proxy representation of soil moisture. The term ‘volumetric water content’ refers to the numerical assessment of soil moisture expressed as the ratio of water volume to soil volume (Abebrese et al., 2024). Representing soil moisture using volumetric water content has been made in previous studies, including studies of sandy loam soils (Abebrese et al., 2024; Cao et al., 2020; Grégoire et al., 2022; Kim et al., 2020; Liu et al., 2023). For instance (Cao et al., 2020) used the volumetric water content as a measure of soil moisture after intense rainfall, and found that 89% of the variation in VSWC is likely explained by the dielectric constants in the soil depth range of 0–40 cm.

In the current study, FieldScout TDR 350 (Spectrum Technologies, Aurora, IL) was used to measure soil volumetric water content (measured in %). Two 20 cm stainless steel probes were inserted vertically to a depth of 20 cm at the randomly selected spots. Such a measure and depth of measurement (20 cm) have been carried out in a previous study (see e.g., Walker & Powell, 2001). The TDR 350 equipment has been used to take in situ measurements of soil volumetric water content in previous studies (see e.g., Akbari et al., 2024; Cheng et al., 2024; Doherty et al., 2024). For example, a previous study by Akbari et al. (2024) assessed the relationship between gravimetric measurements and TDR 350 and noted a strong agreement between them in terms of moisture measurements (R^2^ = 0.96). In another study (see Cheng et al., 2024), drought indices were constructed to characterize soil moisture in winter wheat fields using unmanned aerial vehicles (UAVs) equipped with LiDAR, thermal infrared, and multispectral sensors. Cheng et al. (2024) found that the Improved Temperature–Vegetation Drought Index (ITNDVI) would likely effectively characterize soil moisture content, with the ITNDVI-characterized soil moisture showing a significant correlation with measured surface soil moisture content.

The DEM shows the elevation of the landscape, and its unit is meters. Slope, aspect (including south and north-facing aspect), and topographic wetness index (TWI) were calculated in ArcGIS (ArcGIS Pro) based on the DEM. Slope, measured in degrees, is the steepness of the earth’s surface. Aspect, measured in degrees, is the direction of the slope. TWI measures how topography influences the hydrological flow and wetness of the landscape, and as an index, this variable is unitless; higher numbers mean a wetter surface or slope, and lower numbers mean vice versa. Variables such as slope, aspect, and TWI were included based on their inclusion in previous research (see Iverson et al., 2004; Kopeckỳ et al., 2021; Raduła et al., 2018; Winzeler et al., 2022), even though this is a hypothesis-based study involving the relationship between grasses and shrubs on soil moisture.

### Variables and models

In this study, as noted earlier, the goal was to model the relationship between soil moisture (represented by volumetric water content), open grasses, open shrubs, closed shrubs, and other control variables. Here, the dependent variable is soil moisture (%). Soil moisture is measured as a continuous variable. Independent variables, including the presence of bare land, closed shrubs, open shrubs, and open grasses, were used in the model. These four independent variables were entered as dummy variables where the presence of bare land, open shrubs, closed shrubs, and open grasses is represented by 1, and absence is 0. Additional variables tested include slope, north-facing direction, south-facing direction, TWI, elevation, and temperature of soil (measured in degrees Celsius), and these were entered as independent variables. Slope and elevation information were entered as continuous, but north-facing and south-facing directions were entered as dummy variables.

Generalized linear equations executed in ArcGIS Pro 3.3 were specified as follows. Both univariate and multivariable linear equations were used to express the relationship between soil moisture and the independent variables considered in the study. For the univariate models, independent variables, including bare land, open grasses, open shrubs, closed shrubs, elevation, slope, TWI, north-facing direction, south-facing direction, and soil temperature, were each tested independently against soil moisture (See Equations [Eqns.] 1–10). Running a univariate model on every possible independent variable was done to highlight the relative strengths of the independent variables in predicting soil moisture and also how their predictive performance would differ in multivariable models.

For the multivariable model, we first established a relationship between soil moisture and independent variables, including vegetation cover types, slope, elevation, north-facing direction, south-facing direction, TWI, and temperature of soil (Eq. 11). Second, another multivariable model was built to include soil moisture and any statistically significant variables, including open grasses, open shrubs, and closed shrubs (Eq. 12). The second multivariable model was built to improve Eq. 12 parameters, including coefficients and standard errors, and thus to produce a more robust model. The outcome of the models and the relationships have been highlighted in the results and discussion sections.

1 $$\mathrm{g}\left({\mathrm{SM}}_{\mathrm{i}}\right)={\upbeta }_{0}+{\upbeta }_{1}{\mathrm{BAL}}_{\mathrm{i}}{ +\upphi }_{\mathrm{i}}$$

2 $$\mathrm{g}\left({\mathrm{SM}}_{\mathrm{i}}\right)={\upbeta }_{0}+{\upbeta }_{1}{\mathrm{OG}}_{\mathrm{i}}+{\upphi }_{\mathrm{i}}$$

3 $$\mathrm{g}\left({\mathrm{SM}}_{\mathrm{i}}\right)={\upbeta }_{0}+{\upbeta }_{1}{\mathrm{OS}}_{\mathrm{i}}{+\upphi }_{\mathrm{i}}$$

4 $$\mathrm{g}\left({\mathrm{SM}}_{\mathrm{i}}\right)={\upbeta }_{0}+{\upbeta }_{1}{\mathrm{CS}}_{\mathrm{i}} {+\upphi }_{\mathrm{i}}$$

5 $$\mathrm{g}\left({\mathrm{SM}}_{\mathrm{i}}\right)={\upbeta }_{0}+ {{\upbeta }_{1}{\mathrm{EL}}_{\mathrm{i}}+\upphi }_{\mathrm{i}}$$

6 $$\mathrm{g}\left({\mathrm{SM}}_{\mathrm{i}}\right)={\upbeta }_{0}+{\upbeta }_{1}{\mathrm{SL}}_{\mathrm{i}} {+\upphi }_{\mathrm{i}}$$

7 $$\mathrm{g}\left({\mathrm{SM}}_{\mathrm{i}}\right)={\upbeta }_{0}+{{\upbeta }_{1}{\mathrm{TWI}}_{\mathrm{i}}+\upphi }_{\mathrm{i}}$$

8 $$\mathrm{g}\left({\mathrm{SM}}_{\mathrm{i}}\right)={\upbeta }_{0} {+{\upbeta }_{1}{\mathrm{NF}}_{\mathrm{i}}+\upphi }_{\mathrm{i}}$$

9 $$\mathrm{g}\left({\mathrm{SM}}_{\mathrm{i}}\right)={\upbeta }_{0}+{\upbeta }_{1}{\mathrm{SF}}_{\mathrm{i}}{+\upphi }_{\mathrm{i}}$$

10 $$\mathrm{g}\left({\mathrm{SM}}_{\mathrm{i}}\right)={\upbeta }_{0}+{\upbeta }_{1}{\mathrm{ST}}_{\mathrm{i}}{+\upphi }_{\mathrm{i}}$$

11 $$\mathrm{g}\left({\mathrm{SM}}_{\mathrm{i}}\right)={\upbeta }_{0}+{\upbeta }_{1}{\mathrm{OG}}_{\mathrm{i}1}+{\upbeta }_{2}{\mathrm{OS}}_{\mathrm{i}2}+{\upbeta }_{3}{\mathrm{CS}}_{\mathrm{i}3}+{\upbeta }_{4}{\mathrm{SL}}_{\mathrm{i}4}+ {{\upbeta }_{5}{\mathrm{EL}}_{\mathrm{i}5}+ {\upbeta }_{6}{\mathrm{TWI}}_{\mathrm{i}6}+{\upbeta }_{7}{\mathrm{NF}}_{\mathrm{i}7}+{\upbeta }_{8}{\mathrm{SF}}_{\mathrm{i}8}+{\upbeta }_{9}{\mathrm{ST}}_{\mathrm{i}9}+\upphi }_{\mathrm{i}}$$

12 $$g\left({\mathrm{SM}}_{i}\right)={\beta }_{0}+{\beta }_{1}{\mathrm{OG}}_{i1}+{\beta }_{2}{\mathrm{OS}}_{i2}+{\beta }_{3}{\mathrm{CS}}_{i3}+{\phi }_{i}$$

where $g\left({SM}_{i}\right)$ is the function for soil moisture (SM), the dependent variable, at location *i;* ${\beta }_{0}$ is the intercept or constant or baseline value for the dependent variable, SM, at location *i;* BAL is bare land at location *i;* OG is open grasses at location *i*; OS is open shrubs at location *i*; CS is closed shrubs at location *i*; SL is slope at location *i*; EL is elevation at location *i*; TWI is the topographic wetness index at location *i*; NF is north-facing direction at location *i*; SF is south-facing direction at location *i*; ST is soil temperature measured at location *i*; and *ϕ* is the stochastic error term related to measurements of variables at location *i.*

### Model robustness and multi-collinearity tests

The robustness of the model was determined by using multiple methods to ensure data fit the model. First, the coefficient of determination (also known as R-squared, R^2^) was used to determine how much of the independent variables explain or account for the variance in the dependent variable. The higher the R^2^ value, the more robust the model is, and vice versa. However, to ensure further robustness, the p-value of the R-squared value should be statistically significant. In this study, for the measured coefficient of determination to be statistically significant, the p-value should be less than 0.05. Second, to ensure every independent variable contributes significantly to explaining the variation in the dependent variable, we used the variance inflation factor (VIF) to check for multicollinearity. That is, we determined that no independent variables (two or more) serve the same purpose or present the same effect. Variance inflation, as the name suggests, occurs when the variance in the coefficient of a model is inflated above what it would be if the R^2^ is equal to zero (O’brien, 2007). This variance inflation would occur due to a high correlation between two or more independent variables in the equation. A regression equation’s multicollinearity level can be accepted if the VIF value is less than 10, but for more stringent mindfulness of multicollinearity, a VIF less than 4.0 is recommended (O’brien, 2007). Spatial autocorrelation based on Moran’s I was used to determine whether or not the residual or errors in the output of the regression analysis were random, clustered, or dispersed. It is important to note that Moran’s I evaluates the locations of features and their associated attribute to determine whether they are clustered, dispersed, or randomly distributed.

## Results

### Univariate analysis

**Fig. 3:**
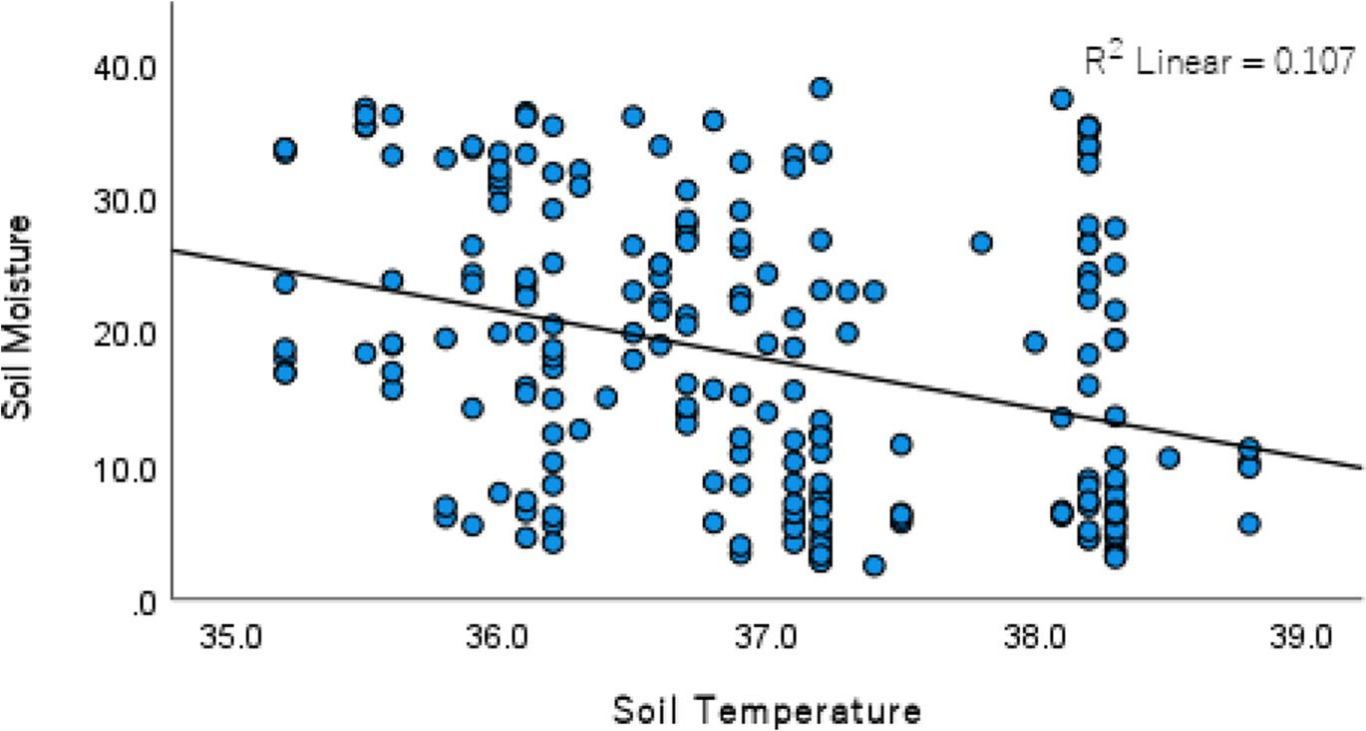
A scatter plot showing the distribution of points explaining the relationship between soil moisture and soil temperature [Note: R^2^ on the scatter plot is 0.017, but the adjusted R^2^ is 0.103]

Our results from the univariate analysis show different strengths and directions of relationships between independent variables and soil moisture assessed in the study. The analysis revealed a significant negative relationship between the presence of bare land and soil moisture (Table 1). It is important to note that relationships between soil moisture and the dummy independent variables are not meaningfully expressed statistically in scatter plots. Thus, a scatter plot showing the relationship between soil moisture and the non-dummy (continuous) independent variable of soil temperature is shown in Fig. 3.

**Table 1:** Univariate linear regression results predicting soil moisture based on Eqs. 1–10

Variable	Constant/Intercept	Coefficient (B)	Standard Error	t-Statistic	p-value	R-squared	Adjusted R-squared
Bare	24.296	−15.464	1.019	−15.181	0.001**	0.518	0.516
Open Grass	16.953	9.912	2.345	4.227	0.001**	0.076	0.072
Open Shrub	15.631	8.757	1.525	5.743	0.001**	0.131	0.127
Closed Shrub	16.320	6.816	1.635	4.169	0.001**	0.074	0.070
Elevation	51.275	−0.199	0.526	−0.378	0.706	0.001	−0.004
Slope	15.761	1.048	0.943	1.115	0.266	0.006	0.001
TWI	16.315	0.189	0.423	0.446	0.656	0.001	−0.004
North-Facing Direction	17.652	3.287	2.676	1.199	0.232	0.007	0.002
South-Facing Direction	17.267	0.040	1.888	0.021	0.982	0.000	−0.005
Soil Temperature	152.643 (17.534^**+**^)	−3.643	0.712	−5.117	0.001**	0.107	0.103

** indicates p-value less than 0.01; the p-value is considered significant when it is less than 0.05, and TWI is the topographic wetness index

Note: ^+^ In our data, soil temperatures range from 25.56 °C to 47.78 °C and cannot be assumed to be 0 °C. Also, logically, soil moisture cannot be more than 100%. Consequently, saying that when the temperature is 0 °C, soil moisture is 152.643% would have very limited real-world application. To provide a more interpretable constant (intercept) for the univariate regression model predicting soil moisture from soil temperature, the intercept or constant was recalculated to represent the predicted soil moisture at the mean observed soil temperature (37.07 °C, based on our data). This approach, known as centering the predictor variable, allows for a more meaningful interpretation of the intercept within the observed data range (Cohen, 2003; Field, 2024). The recalculated intercept is 17.534%, representing the predicted soil moisture when the soil temperature is at its mean. The new intercept calculation is given as New Intercept = Original Intercept + (Original Coefficient of Soil Temperature × Mean of Soil Temperature).

The analysis conveyed that the presence of bare land explains 51.6% of the variation in soil moisture. The presence of bare land is associated with a decrease in soil moisture by 15.464% compared to areas that are not bare. With a constant value of 24.296 associated with bare land, the study found that areas that are not bare (open grasses, open shrubs, and closed shrubs) would have soil moisture of 24.296% (Table 1; also see Eq. 13—Eq. 15).

13 $$\mathrm{g}\left({\mathrm{SM}}_{\mathrm{i}}\right)=24.296-15.464{ (\mathrm{BAL}}_{\mathrm{i}})$$

14 $$\text{For BAL presence},\text{ the model predicts g}\left({\mathrm{SM}}_{\mathrm{i}}\right)=24.296-15.46 \left(1\right)=8.832 \%$$

15 $$\text{For BAL absence},\text{ the model predicts g}\left({\mathrm{SM}}_{\mathrm{i}}\right)=24.296-15.46 \left(0\right)=24.296\%$$

The analysis revealed a significant positive relationship between the presence of open grasses and soil moisture (Table 1). The study found that the presence of bare land explains 7.6% of the variation in soil moisture. The presence of open grasses increases soil moisture by 9.912% compared to areas that do not have open grasses. With a constant value of 16.953 associated with open grasses, the study found that areas that are not open grasses (bare land, open shrubs, and closed shrubs) would have a soil moisture of 16.953% (Table 1; also see Eq. 16 – Eq. 18).

16 $$\mathrm{g}\left({\mathrm{SM}}_{\mathrm{i}}\right)=16.953+{9.912 (\mathrm{OG}}_{\mathrm{i}})$$

17 $$\text{For the presence of open grasses},\text{ the model predicts g}\left({\mathrm{SM}}_{\mathrm{i}}\right)=16.953+9.912 \left(1\right)=26.865\%$$

18 $$\text{For the absence of open grasses},\text{ the model predicts g}\left({\mathrm{SM}}_{\mathrm{i}}\right)=16.953+9.912 \left(0\right)=16.953\%$$

The results revealed a significant positive relationship between open shrubs and soil moisture (Table 1). The presence of open shrubs explains 13.1% of the variance in soil moisture. The presence of open shrubs increases soil moisture by 8.757% compared to areas that do not have open shrubs. With a constant value of 15.631 associated with open grasses, the study found that areas that do not have open shrubs (bare land, open grasses, and closed shrubs) would have a soil moisture of 15.631% (Table 1; also see Eq. 19 – Eq. 21).

19 $$\mathrm{g}\left({\mathrm{SM}}_{\mathrm{i}}\right)=15.631+8.757{(\mathrm{OS}}_{\mathrm{i}})$$

20 $$\text{For the presence of open shrubs},\text{ the model predicts g}\left({\mathrm{SM}}_{\mathrm{i}}\right)=15.631+8.757 \left(1\right)=24.388\%$$

21 $$\text{For the absence of open shrubs},\text{ the model predicts g}\left({\mathrm{SM}}_{\mathrm{i}}\right)=15.631+8.757 \left(0\right)=15.631\%$$

The analysis revealed a significant positive relationship between the presence of closed shrubs and soil moisture (Table 1). The study found that the presence of closed shrubs accounts for 7% of the variance in soil moisture, as indicated by the adjusted R-squared value. The presence of closed shrubs increases soil moisture by 6.816% compared to areas without closed shrubs. With a constant value of 16.320 associated with closed shrubs, the analysis revealed that areas that do not have closed shrubs (bare land, open shrubs, and open grasses) would have a soil moisture of 16.320% (Table 1; also see Eq. 22 – Eq. 24).

22 $$\mathrm{g}\left({\mathrm{SM}}_{\mathrm{i}}\right)=16.320+6.816 {(\mathrm{CS}}_{\mathrm{i}})$$

23 $$\text{For the presence of closed shrubs},\text{ the model predicts g}\left({\mathrm{SM}}_{\mathrm{i}}\right)=16.320+6.816 \left(1\right)=23.136\%$$

24 $$\text{For the absence of closed shrubs},\text{ the model predicts g}\left({\mathrm{SM}}_{\mathrm{i}}\right)=16.320+6.816 \left(0\right)=16.320\%$$

The results of our analysis revealed a significant negative relationship between the presence of soil moisture and elevation, but the relationship is not statistically significant (Table 1). Slope, TWI, north-facing direction, and south-facing direction have positive relationships with soil moisture, but similar to the elevation data, these univariate positive relationships are not statistically significant (Table 1). The results of the analysis revealed a significant negative relationship between soil temperature and soil moisture (Table 1, also see Fig. 3). The study found that soil temperature explains 10.3% of the variation in soil moisture. A unit increase in temperature is associated with a 3.643% decrease in soil moisture. With a constant value of 17.534 associated with soil temperature, the outcome of our analysis show that soil moisture would be 17.534% when soil temperature is at its mean value (37.07 °C) (Table 1; also see Eq. 25).

25 $$\mathrm{g}\left({\mathrm{SM}}_{\mathrm{i}}\right)=17.534-3.643({\mathrm{ST}}_{\mathrm{i}}-\text{Mean }{\mathrm{ST}}_{\mathrm{i}})$$

**Fig. 4:**
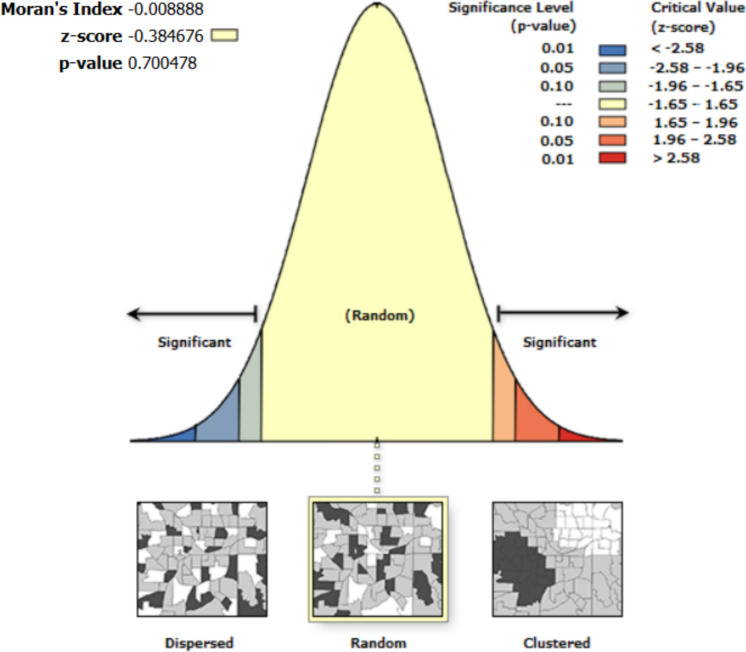
Moran’s I autocorrelation analysis output for soil moisture and bare land [Note: Given the z-score of −0.666, the pattern does not appear to be significantly different than random; Moran's Index = −0.003, expected index = −0.005, variance = 0.000, z-score = −0.666, p-value = 0.505

**Fig. 5:**
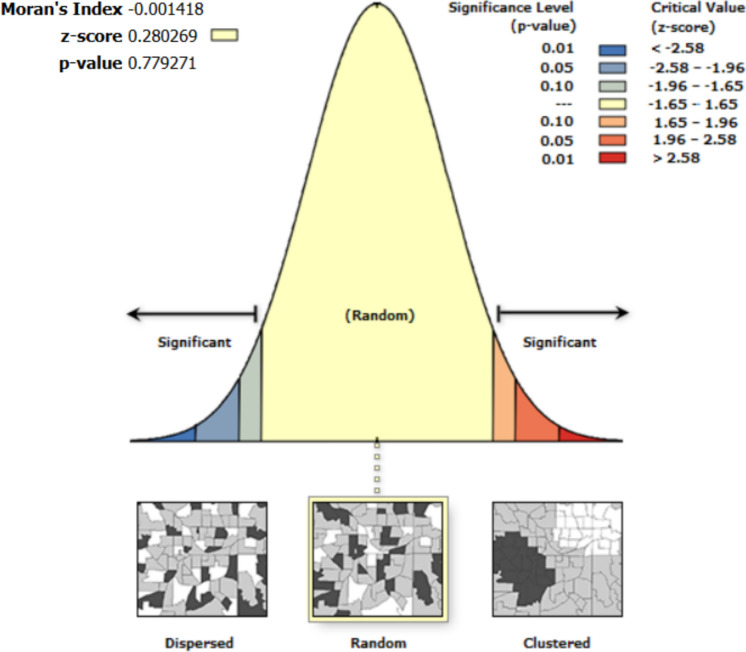
Moran’s autocorrelation analysis output for soil moisture and closed shrubs [Note: Given the z-score of 0.280, the pattern does not appear to be significantly different than random; Moran's Index = −0.001, expected index = −0.005, variance = 0.000, z-score = 0.280, p-value = 0.779

**Fig. 6:**
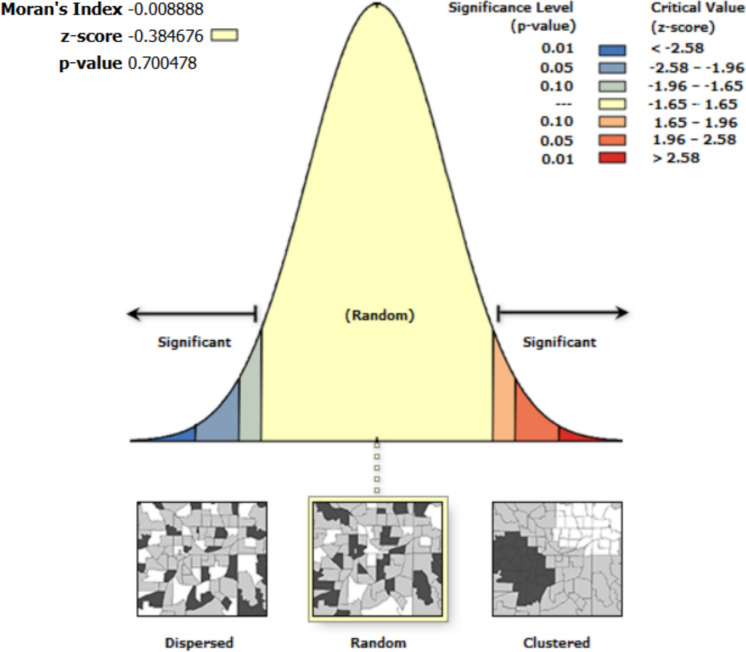
Moran’s I autocorrelation analysis output for soil moisture and open grasses [Note: Given the z-score of −0.385, the pattern does not appear to be significantly different than random; Moran's Index = −0.009, expected index = −0.005, variance = 0.000, z-score = −0.385, p-value = 0.700

**Fig. 7:**
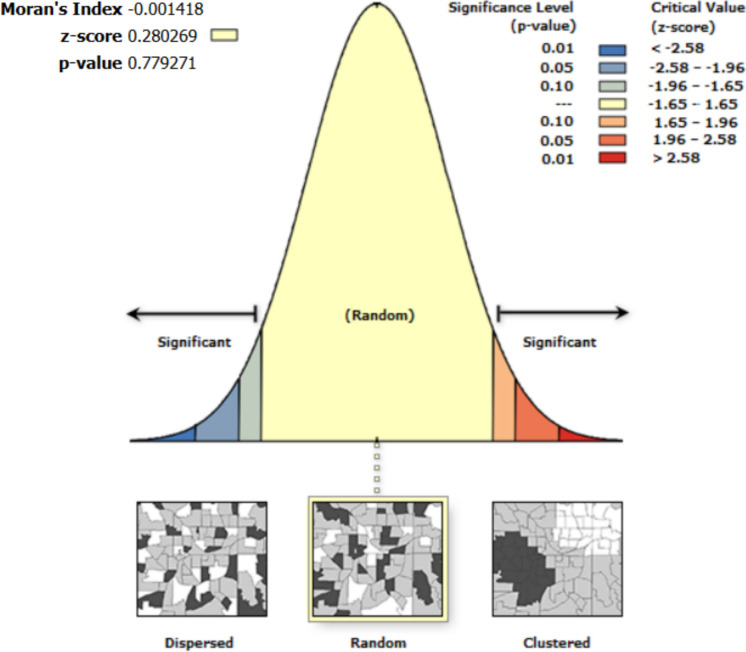
Moran’s I autocorrelation analysis output for soil moisture and open shrubs [Note: Given the z-score of −1.250, the pattern does not appear to be significantly different than random; Moran's Index = −0.019, expected index = −0.005, variance = 0.000, z-score = −1.250, p-value = 0.211

**Fig. 8:**
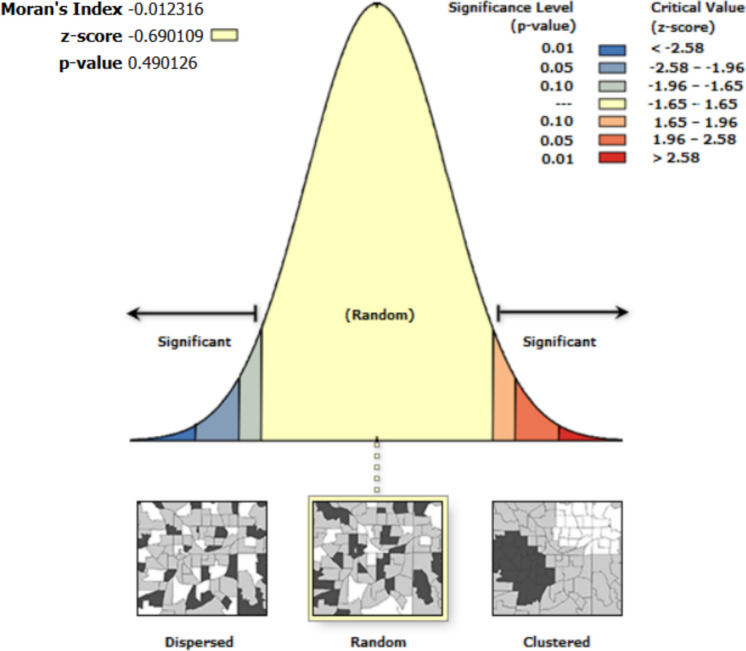
Moran’s I autocorrelation analysis output for soil moisture and soil temperature. [Note: Given the z-score of −0.690, the pattern does not appear to be significantly different than random; Moran's Index = −0.012, expected index = −0.005, variance = 0.000, z-score = −0.690, p-value = 0.490]

Spatial autocorrelation results show that the residuals from the regression are random. Thus, the errors and residuals among variables in the univariate analysis are not spatially interdependent (Figs. 4, 5, 6, 7, 8).

### Multivariable analysis

For the multivariable analysis, the results show two major model outcomes. The first model included variables, elevation, slope, topographic wetness index, north-facing direction, south-facing direction, and soil temperature, in addition to the presence of open grasses, open shrubs, and closed shrubs. The results of Eq. 11 show that the independent variables, including open grasses, open shrubs, closed shrubs, elevation, slope, topographic wetness index, north-facing direction, south-facing direction, and soil temperature, explain approximately 53% of the variance in soil moisture (see Table 2).

**Table 2:** Predicted relationship between soil moisture, vegetation types, and other variables in Eq. 11

Model	R	R Square	Adjusted R Square
Model Summary Based on Eq. 11	0.741	0.547	0.527
Predictors: (Constant), open grass, open shrub, closed shrub, elevation, slope, topographic wetness index, north-facing direction, south-facing direction, and soil temperature			

Further results based on Eq. 11 show that independent variables other than vegetation types are not statistically significant. That is, elevation, slope, TWI, north-facing direction, south-facing direction, and soil temperature in Eq. 11 are not statistically significant (Table 3). A constant or intercept value of −32.041 is not statistically significant (Table 3). A standard error of 97.58 of the model’s constant was found to be larger than the coefficient associated with the constant. Nonetheless, standard errors associated with the variables that are not statistically significant are either higher or slightly lower than their coefficient values. More importantly, soil temperature maintained a negative relationship with soil moisture. However, despite showing significant relationships with soil moisture in the univariate regression model (refer to Eq. 25 and Table 1), it appeared not to be statistically significant in the multivariable model (see Table 3).

**Table 3:** Multivariable linear regression results predicting soil moisture based on Eq. 11

Variables in Eq. 11	Coefficients	Standard. Error	t	p-value	VIF
(Constant)	−32.041	97.583	−0.328	0.74	
Open Grasses	18.021	1.811	9.953	0.000**	1.170
Open Shrubs	14.884	1.427	10.432	0.000**	1.610
Closed Shrubs	13.294	1.451	9.165	0.000**	1.551
Elevation	0.375	.502	0.747	0.460	1.940
Slope	1.883	1.035	1.819	0.071	2.562
TWI	0.464	.568	.818	0.411	3.812
North-Facing Direction	2.390	2.445	.978	0.332	1.760
South-Facing Direction	1.824	1.610	1.133	0.261	1.681
Soil Temperature	−0.841	.671	−1.254	0.211	1.681

** p-value is less than 0.01; relationship is significant when p-value < 0.05; VIF is variable inflation factor

The results show that soil moisture measured in/under open grasses is 18.021% higher than the soil moisture measured on bare land. Also, the study found that soil moisture measured under/in open shrubs is 14.882% higher than soil moisture measured on bare land. Soil moisture measured under/in closed shrubs is 13.291% higher than soil moisture measured on bare land. Soil temperatures would likely influence how dry or wet the soil is. However, we found that a unit increase in soil temperature reduces soil moisture by 0.841%, but this outcome was not statistically significant. The VIF values for all the variables, regardless of whether or not they are statistically significant, are less than 10, and most importantly, they are less than 4. With that being noted, the vegetation-related (open grasses, open shrubs, and closed shrubs) variables had lower VIF values compared to the non-vegetation variables in the equation (see Table 3). It can thus be noted that statistically significant variables have lower VIF values compared to the variables that are not statistically significant.

Whereas Eq. 11 tested the relationship between soil moisture and all listed independent variables, Eq. 12 tested the relationships between soil moisture and vegetation types. However, it is important to point out that, based on the results of Eq. 11, only vegetation-related variables have a significant relationship with soil moisture. Thus, Eq. 12 results demonstrate how only the significant independent variables impact the coefficients, the p-value, VIF, and the t-test statistic. The results show that Eq. 12 is different from Eq. 11 as follows. Based on Eq. 12, independent variables (closed shrubs, open grasses, and open shrubs) explained 52% of the variance in the dependent variable (soil moisture) (Table 4), but in Eq. 11, the independent variables explained 53% of the variation in soil moisture. Based on Eq. 12 results, the standard error (0.771) of the constant is less than the coefficient (8.833) of the constant (see Table 5). However, the standard error of the constant is higher than the coefficient of the constant, based on the results of Eq. 11 (Table 3). Reasons for these differences are highlighted in the discussion section.

**Table 4:** Predicted relationship between soil moisture and vegetation-related variables based on Eq. 12

Description	R	R Square	Adjusted R Square
Model summary based on Eq. 12	0.731	0.526	0.520

*Predictors: (constant), closed shrub, open grass, and open shrub

**Table 5:** Model coefficients Predicted relationship between soil moisture and vegetation-related variables based on Eq. 12

Variables in Eq. 12	Coefficients	Std. Error	t	Sig	VIF
(Constant)	8.833	0.771	11.460	0.001**	-
Open Grasses	18.035	1.780	10.133	0.001**	1.111
Open Shrubs	15.556	1.242	12.526	0.001**	1.210
Closed Shrubs	14.303	1.286	11.123	0.001**	1.201

** p-value is less than 0.01; relationship is significant when p-value < 0.05; VIF is variable inflation factor

The results, in Table 5, show that soil moisture under/in open grasses is 18.035% higher compared to bare land, with all other variables held constant. Also, soil moisture measured under/in open shrubs is 15.556% higher compared to soil moisture measured on bare land, with all other variables held constant. Similarly, soil moisture measured under/in closed shrubs is 14.303% higher than soil moisture measured on bare land, with all other variables held constant (Table 5; also see Eq. 26—Eq. 28). The relationships between soil moisture and open grasses, open shrubs, and closed shrubs are all significant (p-value < 0.05). More importantly, the t-statistic values are significantly higher than zero, and there is significantly lower multicollinearity as shown in the VIF values that are less than 10.

26 $$g\left({\mathrm{SM}}_{i}\right)=8.833+18.035({\mathrm{OG}}_{i1})+15.556({\mathrm{OS}}_{i2})+14.303({\mathrm{CS}}_{i3})$$

For the presence of OG, OS, and CS, soil moisture value is given as

27 $$g\left({\mathrm{SM}}_{i}\right)=8.833+18.035\left(1\right)+15.556\left(1\right)+14.303\left(1\right)=56.727\%$$

For the absence of OG, OS, and CS, soil moisture value is given as

28 $$g\left({\mathrm{SM}}_{i}\right)=8.833+18.035\left(0\right)+15.556\left(0\right)+14.303\left(0\right)= 8.833\%$$

**Fig. 9:**
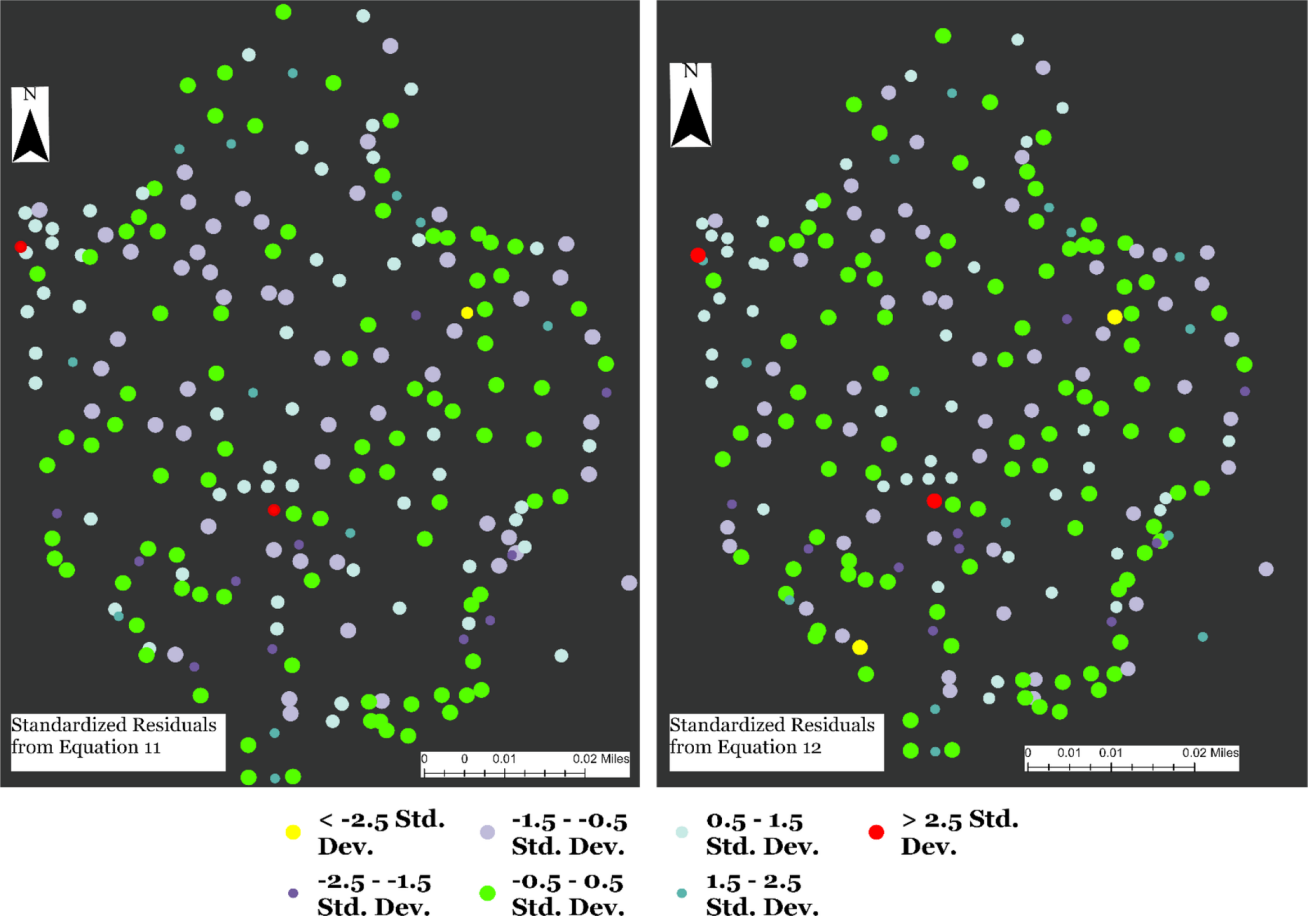
Standardized residuals from Eqs. 11 and 12. Note: In this figure, we show where soil moisture has been overpredicted and underpredicted based on Eqs. 11 and 12. Additionally, the figure illustrates instances where predictions were very close to being accurate, including those that were off by 0.05 or less

The standardized residual values showing how measured soil moisture values deviate from the predicted soil moisture values demonstrate how Eq. 11 compares with Eq. 12. The standardized residuals associated with the outputs from Eqs. 11 and 12 show both similarities and differences between a model and a more robust model. The results show that the highest deviations (> 2.5) in measured soil moisture values occurred at two locations based on predictions from Eqs. 11 and 12. In other words, both models show that at 2 locations, soil moisture was overpredicted by greater than 2.5 (Fig. 9). Thus, in terms of overprediction, the two spatially explicit equations show similar strength. However, Eq. 12 presents a higher number of lowest number of deviations (−0.5 to 0.5) in the predicted soil moisture values compared to Eq. 11. Overall, the lowest deviation in soil moisture predictions occurred at 94 out of 220 locations based on Eq. 12. On the other hand, based on Eq. 11, the lowest deviations in predicted soil moisture values occurred at 88 out of 220 measurement locations.

**Fig. 10:**
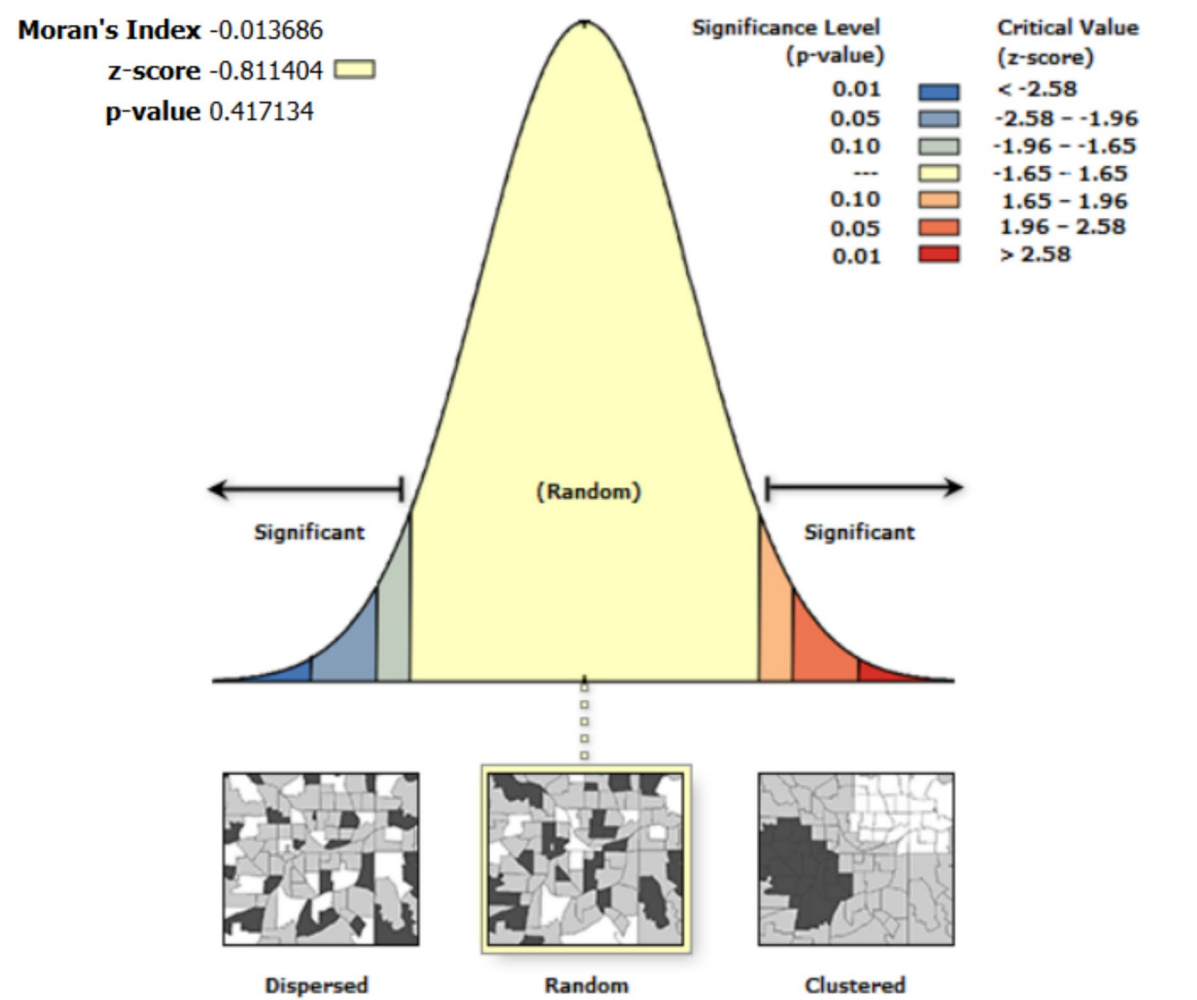
Moran’s I autocorrelation analysis output for Soil Moisture and multiple independent variables (open grasses, open shrubs, closed shrubs, elevation, slope, topographic wetness index, north-facing direction, south-facing direction, and soil temperature). [Note: Given the z-score of −0.811, the pattern does not appear to be significantly different than random; Moran's Index = −0.014, expected index = −0.005, variance = 0.000, z-score = −0.811, p-value = 0.417]

**Fig. 11:**
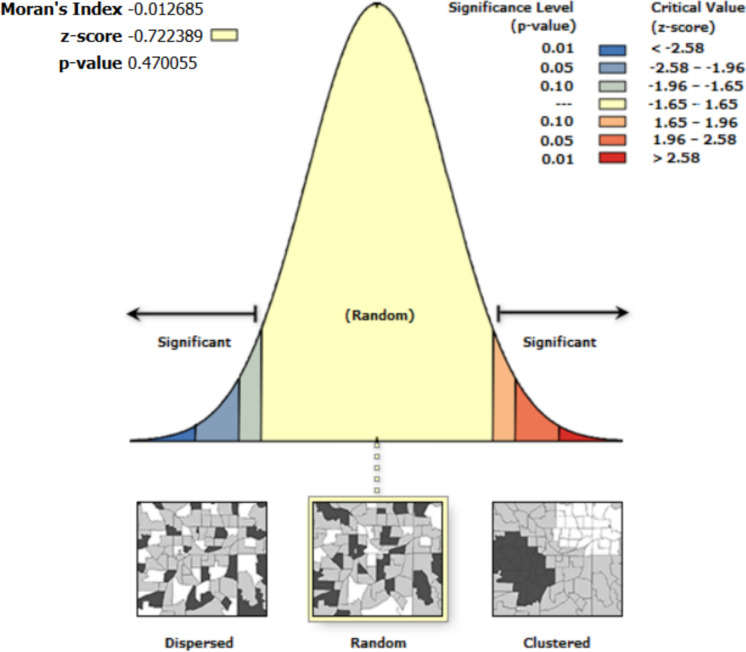
Moran’s I autocorrelation analysis output for Soil Moisture and multiple independent variables (open grasses, open shrubs, and closed shrubs). [Note: Given the z-score of −0.722, the pattern does not appear to be significantly different than random; Moran's Index = −0.013, expected index = −0.005, variance = 0.000, z-score = −0.722, p-value = 0.470]

A spatial autocorrelation report on the standardized residuals of Eq. 11 shows that, given the z-score of 1.54, the pattern does not appear to be significantly different than random (p-value = 0.124). Similarly, given the z-score of 0.16, the pattern of the standardized residuals from Eq. 12 does not appear to be significantly different than random (p-value = 0.88). In accounting for standardized residuals not being statistically different from random, the p-value for Eq. 12 is higher (0.88) than the p-value (0.12) of Eq. 11. However, given that these are not significantly different from random distribution, overall, these results show that errors or deviations of the predicted soil moisture values from the measured soil moisture values are randomly distributed across the geographical space where this study took place. Most importantly, the outcome follows statistical independence in such a way that errors in measurements or predictions at one location do not depend on errors from other locations across space. Spatial autocorrelation results based on the multivariable models (Eq. 11 and Eq. 12) show that the standardized residuals (Fig. 9) show that the errors are random. Figures 10 and 11 show the spatial autocorrelation results.

## Discussions

### Main findings

This hypothesis-based study was such that there is no significant relationship between soil moisture and open grasses, open shrubs, and closed shrubs. Even though this is a hypothesis-based study, it presents important insights regarding how much variation in soil moisture is explained by the vegetation types. The final model based on Eq. 12 demonstrates that vegetation cover types moderately explain variation in soil moisture across the landscape (Dai et al., 2022; Guo et al., 2020; Wang et al., 2012a). As noted in a previous study (see Ghulam et al., 2011; Marshall et al., 2021; Millard et al., 2018; Venkatesh et al., 2011), vegetation types alone cannot account for the amount of soil moisture, and there are several parameters to test and see how well they predict soil moisture. Thus, it is important to note that having a hypothesis-based study is a starting point for surveying a lot more variables that would likely relate to soil moisture, especially in a savanna landscape. In an initial model, several other variables apart from vegetation types were added to test how these variables would contribute to the model and even improve how vegetation cover types synergize to effectively predict soil moisture (see van Oorschot et al., 2023). The twelfth model involved only soil moisture and vegetation types. The results of models 11 and 12 show that there is a significant relationship between soil moisture, open grasses, open shrubs, and closed shrubs. Previous studies have had similar results (see He, 2014; Pockman & Small, 2010; Traff et al., 2015). Thus, the initial (null) hypothesis of the study is rejected based on the findings. One major finding of this study is the significant negative relationship observed between soil moisture and soil temperature at the univariate level. However, the relationship is rendered insignificant at the multivariable level when other independent variables (open grasses, open shrubs, closed shrubs, elevation, slope, TWI, north-facing direction, and south-facing direction) are introduced. While soil temperature would likely have a fundamental control on soil moisture (as suggested by the univariate analysis), the multivariable model based on Eq. 11 suggests that the presence of vegetation cover types, elevation, and slope provides the most significant unique linear explanations for soil moisture variability. So, the linear power of these variables in predicting soil moisture masks the effect of soil temperature. Additionally, the information on soil temperature would likely be redundant because of the higher linear relationship between soil moisture, open grasses, open shrubs, and closed shrubs. Hence, this study presents an open hypothesis (vegetation cover types would likely mask the predictive power of temperature in modeling soil moisture) that could be further tested in future investigations.

### Interpretation of findings in the context of previous studies and empirical contribution

This study presents both methodological and empirical insights. Empirically, this study has presented significant insights into the distinction between the various vegetation types (open grasses, open shrubs, and closed shrubs, compared to a reference variable (bare land)), in terms of their relationship with soil moisture. Our study findings imply that soil moisture is higher under all three savanna vegetation types (open shrubs, open grasses, and closed shrubs) compared to bare land, and this is supported by previous studies (see Cheng et al., 2020; Dai et al., 2022; De Souza et al., 2021; Zhou et al., 2019). Further, the findings of this study suggest that open grasses, open shrubs, and closed shrubs have high, higher, and highest amounts of soil moisture, respectively, when comparing how each of them is compared to soil moisture on bare land. Whereas there are no studies in the study area to compare these findings with, especially in tropical savanna regions, there are previous studies in other tropical regions to compare the findings of the current study with. For instance, the finding of the current study is similar to the outcome of a previous study on the Loess Plateau in China, which noted that sub-shrub and grass sites exhibit a higher soil moisture content due to their greater soil retention capacity, especially during the dry season (see Wang et al., 2012a, 2012b). Also, in south-west China, it has been found that soil moisture reduces by about 32.1% when vegetation converts to bare land and by 70% when deforestation leads to rock desertification (see Chen et al., 2009).

The results of the current study imply that soil moisture under all the vegetation-related variables, especially grasses, is higher than soil moisture on bare land. In a similar study in the Western Ghats in India, there were significant differences in soil moisture in terms of differences in depths, but no such significant differences with depth were noticed under acacia and degraded land covers (Venkatesh et al., 2011). However, Venkatesh et al. (2011) did not predict a relationship between vegetation types and soil moisture. A previous study in a semi-arid area in Brazil found that the conversion of Caatinga vegetation into soil surface coverages changes soil moisture distribution patterns, including a reduction in the coefficient effect of vegetation cover on soil moisture by between 26 and 47% (de Queiroz et al., 2020). Such an outcome reiterates the findings of the current study in that, generally, soil moisture under all the vegetation cover types is higher than the soil moisture from bare soil surfaces. Our study findings reinforce how high soil moisture under grasses is, including when compared with other vegetation types on the landscape (Wang et al., 2010). Wang et al. (2010) found that areas of farmland in 1987 had been converted into grassland by 2000, and soil moisture mainly increased, with increases ranging from 20 to 60%. Also, it was noted that from 2000 to 2005, most of the grasslands in the northern part of the Yongding River basin and some grassland in the central area were converted into farmland, and soil moisture decreased by up to 60% (Wang et al., 2010).

### Interpretation of findings in the context of methodological contribution

Methodologically, the study sets up two main multivariable models to assess how soil moisture relates to some variables. The findings of the study imply that there is a clear difference between the two models in terms of their fit to the data. While the first multivariable model had an R^2^ value higher than that of the second multivariable model, several indicators imply that the second model fits the data better and thus is the more robust model. For instance, the eleventh model’s constant has a negative t-test statistic, a characteristic of a model not sufficiently specified. On the other hand, the twelfth model’s constant has a t-test statistic of 11.460, which is positive and significantly higher than zero, and thus shows that it is correctly specified, and it is more robust compared to the eleventh model. The transition from a negative to positive t-test statistic between the eleventh and twelfth models implies that the control independent variables tested alongside the open grasses, open shrubs, and closed shrubs in the eleventh model do not have enough linear predictive power to significantly explain soil moisture. Thus, this study has shown that on a savanna landscape where mining has rendered the soil bare, vegetation cover is necessary to maintain some soil quality, and that vegetation cover types would predict soil moisture significantly more than terrain variables (e.g., slope, aspect, elevation, TWI) and temperature. Even though Eq. 11 output has a higher R-squared value than the Eq. 12 output, the latter has a more modest standard error of estimates for the variables tested. Also, Eq. 12 has more reasonable constant values in general compared to the output of Eq. 11. However, more importantly, the standard error of the constant value of Eq. 12 is also reasonable compared to that of Eq. 11, and thus, Eq. 12 is a more robust model predicting soil moisture.

Additional methodological insights from the current study have been discussed as follows. The study created two multivariable models that present important insights into differences in how soil moisture relates to vegetation and other variables. The findings of the study imply that Eq. 12 is more improved compared to Eq. 11. For example, while Eq. 11 presents a relationship in which standard errors are almost the same or sometimes even higher than the coefficients of the variables, Eq. 12 presents standard errors significantly less than the coefficients of the variables. Also, removing the other variables that are not significant increases the coefficient of vegetation-related variables. That is, the positive effect of the open grasses, open shrubs, and closed shrubs in predicting soil moisture increases with the elimination of the non-significant variables. For instance, the coefficient of open grasses increases by 0.014%; that of open shrubs increases by 0.672%; and that of closed shrubs increases by 1.009%. These methodological insights reinforce the fact that fitting a model that explains the variance in soil moisture and adding terrain or relief variables would not necessarily improve the accuracy or robustness of the model. Nonetheless, terrain variables were tested in this study because they have proven to be relevant in other landscape-related studies, as noted in the methodology section of this article. With this being noted, with our results, we show that fewer independent variables would also likely present a useful and more accurate model.

## Implications for sustainable land management

Soil moisture is very important in improving landscapes where vegetation degradation has occurred, especially in areas where revegetation efforts are challenged by drought and aridity problems, as in any savanna region of the world. The broader maintenance of soil moisture may rely on improving the coverage of grasses on bare land, as indicated by the results of this study, and thus enhancing reclamation efforts and reversing land degradation. Due to the higher soil moisture at the locations with open grasses, conservation and land reclamation efforts, including the planting of trees, may focus on these moisture-laden soils. For areas that already have grass cover, the moisture-laden soil under grass could be the starting point for bringing back the tree- and non-tree shrubs that had been removed because of surface mining activities. In terms of their relevance in ecosystems compared to big trees, “shrubs share nutrients and information with other plants, engage in the carbon-sugars-for-nutrients trade with fungi, shelter microbes in return for nitrogen and other nutrients, and thus contribute to a healthy, biodiverse, carbon-sequestering soil system” (Fisher, 2019, p 1). Our study provides spatial information for improving landscape greenness after mining activities that render vegetated spaces bare. This study specifically points to where moisture-laden soil is found in the landscape and where to target spatially to improve vegetation cover and increase biodiversity after degradation from mining activities.

Based on the findings of this current study, it is known that bare soil has very low amounts of soil moisture compared to the vegetation types, open grasses, open shrubs, and closed shrubs). On the bare ground made up of earth materials from digging holes for surface mining, soil management to improve the nutrient level may be required. However, with knowledge gained from the current study regarding the spatial distribution of soil moisture on the mining-induced degraded landscape, planting grasses similar to the current species of grass in the landscape would be worth it. Land managers may have to plant grass first, get moisture-laden soil under the grass, and follow up with planting tree- and non-tree shrubs. The justification for having shrubs alongside the grasses is that shrubs would likely serve as nurse species, nurture young trees, and that young trees grow better in their company (Fisher, 2019). Whereas grass is needed in the landscape for the reason that the soil under it is moisture-laden, given the current climate change situation, the ultimate goal should be to restore degraded land by re-planting tree- and non-tree shrubs that are native and drought-resistant, which were previously removed due to the mining activities on the landscape. More importantly, land reclamation and conservation efforts should aim at converting the piles of earth materials that are almost barren into a more productive landscape with a mix of short trees and grasses with every species playing an important role in balancing the ecosystem in this era of climate change. With this approach, most degraded landscapes from surface mining could be reversed, and terrestrial ecosystems would be used sustainably. Most importantly, desertification and biodiversity loss, which are characteristics of the landscape in the area, would be combated, and in the long term, Goal 15 of the SDGs would be fulfilled. This study has been able to assess the relationship between soil moisture and vegetation cover types in a hypothesis-based analysis. However, some limiting factors likely impacted the results of the analysis, including biasing the models we created. As noted earlier, access to the mining sites was restricted due to community concern about external monitoring of illegal mining activities. As a result, soil moisture measurements could not be collected across all potential locations, which may limit spatial representativeness. Secondly, the study relied on a single-season field campaign conducted during the rainy season. Soil moisture patterns may differ substantially during the dry season, when vegetation stress and evaporation rates are higher. Finally, the cross-sectional design used in this study did not capture temporal variability in soil moisture or vegetation dynamics; therefore, a longitudinal measurement would offer deeper insights into seasonal or interannual shifts.

## Conclusion

This study focused on establishing a relationship between soil moisture and vegetation cover types in a savanna landscape characterized by a higher proportion of bare land than any other land cover type, resulting from previous surface mining activities. We hypothesized that there is no significant relationship between open grasses, open shrubs, closed shrubs, and soil moisture. Our results indicate that open grasses, open shrubs, and closed shrubs have a positive relationship with soil moisture, and this relationship is statistically significant.

Based on the findings of the study, the following conclusions have been made. First, empirically, this study concludes that there is a significant relationship between vegetation types and soil moisture in this savanna landscape, where topsoil has been highly disturbed by surface mining activities. Also, this study highlights that soil moisture under grasses and shrubs is higher than soil moisture in bare land. We emphasize that while soil temperature can be statistically significant in predicting soil moisture in a univariate model, it is not likely to be significant when other independent variables (e.g., presence of vegetation cover, elevation, slope) are assessed alongside soil temperature. Second, methodologically, even though non-significant independent variables improved the coefficient of determination, eliminating these variables improved the effect of the significant variables on soil moisture. Most importantly, this study concludes that eliminating such independent variables would likely reduce errors and uncertainties associated with predicting soil moisture. While a higher coefficient of determination value is important in terms of how well independent variables explain variation in soil moisture, such higher values would not be reported with enough certainty because of the high standard errors associated with them. Consequently, this study suggests that a more robust model for predicting soil moisture would focus on how independent variables significantly affect soil moisture, as well as significantly reducing errors and uncertainties.

The study findings would likely serve as important information for supporting conservation efforts. For instance, we show that soil moisture is higher among open grasses, open shrubs, and closed shrubs, compared to that of bare land, and thus soil moisture retention strategy for supporting tree growth should begin with the planting of native vegetation and grasses. With this study being hypothesis-based and reporting a moderate value for the coefficient of determination, we suggest that there is a need for exploratory testing of several other independent variables that would also likely predict the amount of moisture. For instance, future studies should likely test independent variables such as humidity and rainfall in addition to the ones tested in this current study. This study was conducted in July, and thus, it is in the rainy season. Consequently, a field survey for data collection during the dry season is recommended to study the relationships between the vegetation types and soil moisture further.

## Data Availability

Data for this manuscript will be made available upon reasonable request.
